# Understanding the positive outcomes of discharge planning interventions for older adults hospitalized following a fall: a realist synthesis

**DOI:** 10.1186/s12877-020-01980-3

**Published:** 2021-01-29

**Authors:** Véronique Provencher, Monia D’Amours, Matthew Menear, Natasa Obradovic, Nathalie Veillette, Marie-Josée Sirois, Marie-Jeanne Kergoat

**Affiliations:** 1 School of Rehabilitation, Faculty of Medicine and Health Sciences, Université de Sherbrooke, Sherbrooke, QC Canada; 2 Research Centre on Aging, Sherbrooke, QC Canada; 3 Department of Family Medicine and Emergency Medicine, Laval University, Québec, Canada; 4 Centre de recherche sur les soins et les services de première ligne, Université Laval, Québec, Canada; 5 School of Rehabilitation, Faculty of Medicine, Université de Montréal, Montréal, QC Canada; 6 Department of Rehabilitation, Faculty of Medicine, Université Laval, Québec, Canada; 7 Department of Medicine, Faculty of Medicine, Université de Montréal, Montréal, QC Canada

**Keywords:** Discharge planning, Falls, Older adults, Transition of care, Review

## Abstract

**Background:**

Older adults hospitalized following a fall often encounter preventable adverse events when transitioning from hospital to home. Discharge planning interventions developed to prevent these events do not all produce the expected effects to the same extent. This realist synthesis aimed to better understand when, where, for whom, why and how the components of these interventions produce positive outcomes.

**Methods:**

Nine indexed databases were searched to identify scientific papers and grey literature on discharge planning interventions for older adults (65+) hospitalized following a fall. Manual searches were also conducted. Documents were selected based on relevance and rigor. Two reviewers extracted and compiled data regarding intervention components, contextual factors, underlying mechanisms and positive outcomes. Preliminary theories were then formulated based on an iterative synthesis process.

**Results:**

Twenty-one documents were included in the synthesis. Four Intervention-Context-Mechanism-Outcome configurations were developed as preliminary theories, based on the following intervention components: 1) Increase two-way communication between healthcare providers and patients/caregivers using a family-centered approach; 2) Foster interprofessional communication within and across healthcare settings through both standardized and unofficial information exchange; 3) Provide patients/caregivers with individually tailored fall prevention education; and 4) Designate a coordinator to manage discharge planning. These components should be implemented from patient admission to return home and be supported at the organizational level (contexts) to trigger knowledge, understanding and trust of patients/caregivers, adjusted expectations, reduced family stress, and sustained engagement of families and professionals (mechanisms). These optimal conditions improve patient satisfaction, recovery, functional status and continuity of care, and reduce hospital readmissions and fall risk (outcomes).

**Conclusions:**

Since transitions are critical points with potential communication gaps, coordinated interventions are vital to support a safe return home for older adults hospitalized following a fall. Considering the organizational challenges, simple tools such as pictograms and drawings, combined with computer-based communication channels, may optimize discharge interventions based on frail patients’ needs, habits and values.

Empirically testing our preliminary theories will help to develop effective interventions throughout the continuum of transitional care to enhance patients’ health and reduce the economic burden of avoidable care.

**Supplementary Information:**

The online version contains supplementary material available at 10.1186/s12877-020-01980-3.

## Background

Falls among older adults are a worldwide public health concern, especially in the context of an aging population [1]. According to the U.S. Centers for Disease Control and Prevention, falls are the leading cause of non-fatal injuries among older adults, and one in ten falls leads to a serious injury, such as a hip fracture or head injury, which requires hospitalization [2]. In Canada, accidental falls were the main cause (81%) of older adults being hospitalized for injury in 2019, which is a 9% increase over the previous year [3]. In the European Union, it is estimated that each year almost two thirds (62%) of older adults visiting emergency departments for fall-related injuries were admitted to hospital [4].

Older adults hospitalized for serious injuries due to a fall are exposed to significant risks of adverse events after discharge, such as a new fall, functional decline, hospital readmission, and emergency visits [5, 6]. Patients hospitalized for a fall are more likely to be readmitted for a fall within 30 days of discharge than non-fall patients (17.4% vs 3.8%) [7]. Many older patients and their families were also dissatisfied with the hospitalization and discharge process [8, 9]. Recent studies reported that between one and two thirds of post-discharge adverse events could have been prevented [10–12], especially through comprehensive discharge planning [13]. The way the discharge is planned and carried out can thus improve patients’ and families’ satisfaction with the process [8, 9, 14–16] and their quality of life [16, 17].

Many interventions have been developed to optimize discharge planning for hospitalized older adults and positive outcomes for them and their families after the discharge home [18–24]. However, they do not all produce the expected effects to the same extent or in the same way since they feature various intervention components that are delivered at different timepoints in the healthcare continuum and in several healthcare settings and target specific subgroups of older adults. Their efficacy may vary depending on the context in which they are implemented and because they generate their outcomes through diverse mechanisms. The precise nature of these intervention components, how they work and in what circumstances has received little attention. A better understanding of the relationships between discharge planning intervention components, contexts, mechanisms and outcomes could lead to a more optimal design of interventions and improved outcomes for older adults and their families.

The general research question was: How do the key components of discharge planning interventions targeting older adults hospitalized after a fall generate their outcomes, and for whom and in what circumstances are these components effective? Specifically, this study aimed to: 1) Identify the key components of discharge planning interventions for older adults hospitalized after a fall and their outcomes; and 2) Develop preliminary theories that improve our understanding of how these intervention components lead to different outcomes (mechanisms) and in what contexts (when, where and for whom) these components are effective. These preliminary theories will represent an important step towards recommendations for decision-makers and clinicians on how to best design and implement discharge planning interventions for older adults hospitalized following a fall.

## Method

A realist synthesis was performed and reported in accordance with the standards issued by RAMESES (Realist And Metanarrative Evidence Syntheses: Evolving Standards) [25, 26]. This method differs from systematic reviews in that it not only examines the effectiveness of interventions but also helps us to understand why and how they produce the expected outcomes by making their underlying assumptions and processes explicit [25, 26]. In this synthesis, we relied on the work of Dalkin and colleagues (2015) to conceptualize intervention components as resources that are introduced into a context and that alter individuals’ reasoning and behaviors [27]. These changes in reasoning and behavior are then integral parts of the mechanisms that give rise to outcomes. Our realist approach will thus help us to produce initial theories that explain how intervention components (I) provide resources that, when introduced into certain contexts (C), activate mechanisms (M) that in turn generate various outcomes (O), i.e. preliminary ICMO theories relevant to discharge planning for older adults hospitalized following a fall.

### Scoping the literature and focusing the review

An initial scoping of the literature on discharge planning interventions for older adults carried out by the research team in 2016–2017 led to the identification of several intervention components common across discharge planning interventions. Recognizing the need to better understand how and why these components worked, the research team pursued a realist synthesis approach and invited several stakeholders to participate. The research team defined the scope of the realist synthesis with input from partners within the Quebec Ministry of Health and Social Services responsible for older adult care policies. There was a shared interest in focusing the review on interventions that could not only reduce rates of hospitalization, but also improve the satisfaction of older adults. Given their increased risk of adverse outcomes, we decided to focus the synthesis on the population of older adults hospitalized after a fall. This choice led to the need to verify the relevance of the previously identified intervention components to this specific population. Finally, based on our initial literature review, we judged it more appropriate and feasible to focus our synthesis on the development of preliminary program theories and to conduct work to define more robust program theories as a later, second phase of the research.

### Searching process and selection of documents

To identify articles or reports that could be helpful to describe the discharge planning intervention components and develop initial program theories, we conducted searches in nine databases (MEDLINE, CINAHL, Ageline, SCOPUS, ProQuest Dissertations & Theses, EBM Reviews, Health Star, Nursing & Allied Health Database, Health Management Database) as well as the grey literature, including library catalogs (BANQ, Santécom, CUBIQ, Germain [IUGM catalog] and other resources (BDSP [public health data bank], Google, Google Scholar and Social Care Online). Manual searches in reference lists of selected articles were also conducted. This search strategy was developed in partnership with an experienced librarian and verified by a second one. Although a set of natural and controlled keywords was targeted (Table 1) based on three main concepts (population: older adults; interventions / follow-up; outcomes / effects), the search strategy was flexible enough to allow for an iterative process involving searching for evidence-based data, as recommended for realist syntheses [28].

**Table 1:** Database search strategy based on three concepts

CONCEPTS		KEYWORDS
1	Population: older adults + [fall-related]	older adult* elder* senior* old* people geriatric patient* older patient* aged[MESH] AND [accidental fall*] [hip fracture*]
2	Interventions / follow-up	transition of care / transitional care continuity of patient care discharge planning patient discharge hospital discharge return to home / returning home post discharge + follow up / support
3	Outcomes / effects	length of stay in hospital readmission to hospital emergency visit admission to an institution fall mortality functional decline cost health care utilization patient health status patient satisfaction carer satisfaction quality of life well-being

### Selection and appraisal of documents

The selection of documents to be included in the realist synthesis was carried out by a single reviewer following a three-step process: 1) title screening; 2) abstract screening; and 3) full-text screening. Documents were considered eligible if the population of interest was older adults (65 years and older) that had been hospitalized following a fall.

They were also eligible if they described any discharge planning intervention or components of these interventions. We excluded studies that described interventions implemented exclusively post-discharge, in emergency departments, or in palliative care (as our focus was the process of discharge planning for hospitalized older adults). Documents not reporting outcomes (e.g. protocols, abstracts of posters) were excluded. Reverse citation searches to capture studies related to these protocols or abstracts were done when it was relevant, but none were found. Consistent with realist methods, we did not exclude studies based on their research design and a wide variety of design types were eligible for inclusion in the synthesis (e.g. clinical trials, observational studies, qualitative studies, etc.). However, we did exclude any documents published in languages other than English or French. In order to consider evidence consistent with current healthcare contexts and circumstances, we only included documents published in the past decade. As the initial database searches were conducted in 2018 and then updated in 2020, articles published before 2008 were excluded.

In addition to these criteria, we used an assessment grid to select documents based on two other criteria: 1) relevance (contribution to development of the theory); and 2) rigor (validity and credibility of the methods used). The ‘relevance’ criterion was applied throughout the selection process whereas the ‘rigor’ criterion was used during the full-text screening process. Documents were relevant if they contributed information about the contexts, mechanisms or outcomes of discharge planning intervention components. One reviewer examined all relevant empirical articles using the Mixed Methods Appraisal Tool (MMAT) [29], a valid and reliable tool suitable for different types of empirical studies (qualitative, quantitative, mixed) to ensure that selected documents met minimal criteria for rigor. No articles were excluded from the realist synthesis based on the rigor. The principal investigator supervised the complete process to ensure adequate selection of relevant documents.

### Data extraction

With respect to data extraction, one reviewer extracted information on data sources (year of publication, authors, study type), population characteristics (diagnosis, comorbidity/frailty, presence of cognitive deficits) and healthcare settings (acute, post-acute, community). To understand evidence-based effects of intervention components in a specific context, the reviewer then extracted and compiled the data in a table using a classification based on key concepts of a realist synthesis, namely intervention components (I), contextual factors (C), underlying mechanisms (M) and outcomes (O). Extracting data based on these concepts enabled the research team to then synthesize the information in order to develop Intervention-Context-Mechanism-Outcome (ICMO) configurations [25–27, 30–33] that reflect our preliminary theories.

### Analysis and formulation of preliminary program theories

**Fig. 1:**
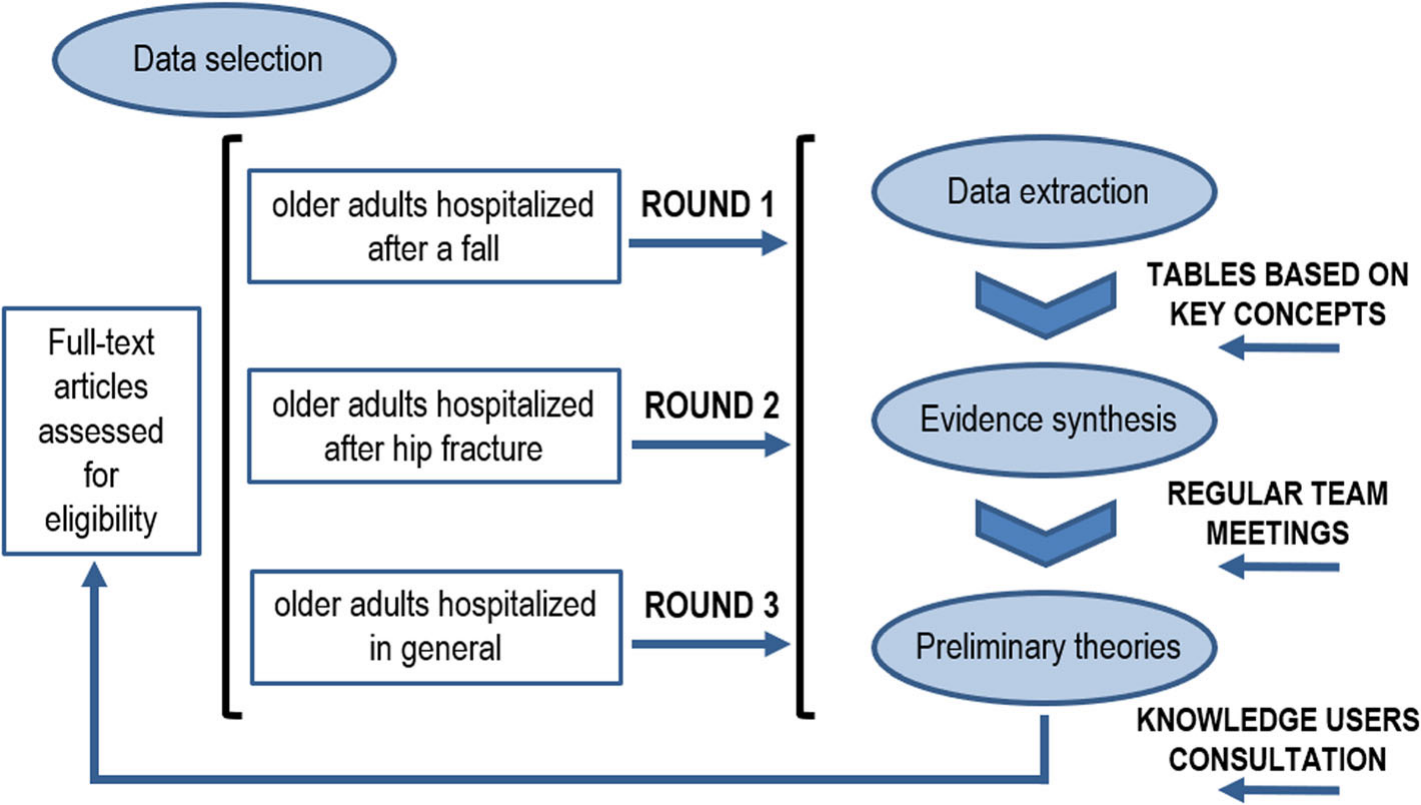
Iterative process used to develop the ICMOs

Two reviewers and the principal investigator then analyzed and synthesized the extracted data as follows: 1) the information gathered was organized by intervention component (I); 2) for each intervention component, we identified recurring patterns of associated outcomes; 3) we examined relationships between underlying mechanisms (M) (i.e. resources, reasoning and behaviors) and specific outcomes (O); and 4) we explored the contextual factors (C) that influenced the expression of M-O relationships. Fig. 1 shows the iterative process we used to develop the ICMOs. Data from articles specifically related to older adults hospitalized after a fall were first analyzed (round 1). The central place of communication, education and coordination in components of discharge planning interventions was highlighted and these became the foundation for developing the preliminary program theories (ICMOs). As there were few articles on discharge planning for older adults hospitalized after a fall that documented contexts and mechanisms, these aspects were enhanced and clarified with complementary sources of data regarding discharge planning for older adults. Full-text assessed for eligibility documents pertaining to hospitalizations for hip fracture (round 2) and in general (round 3) were thus included if they documented mechanisms and contexts and were applicable to fall situations.

Regular team meetings were held throughout the process to discuss emerging ICMO configurations and produce iteratively revised versions. Synthesis of the evidence led to the development of the main preliminary theories resulting from the analysis process. Knowledge users (decision-makers, clinicians) were consulted during this process to ensure that the preliminary theories formulated were clinically relevant.

## Results

**Fig. 2:**
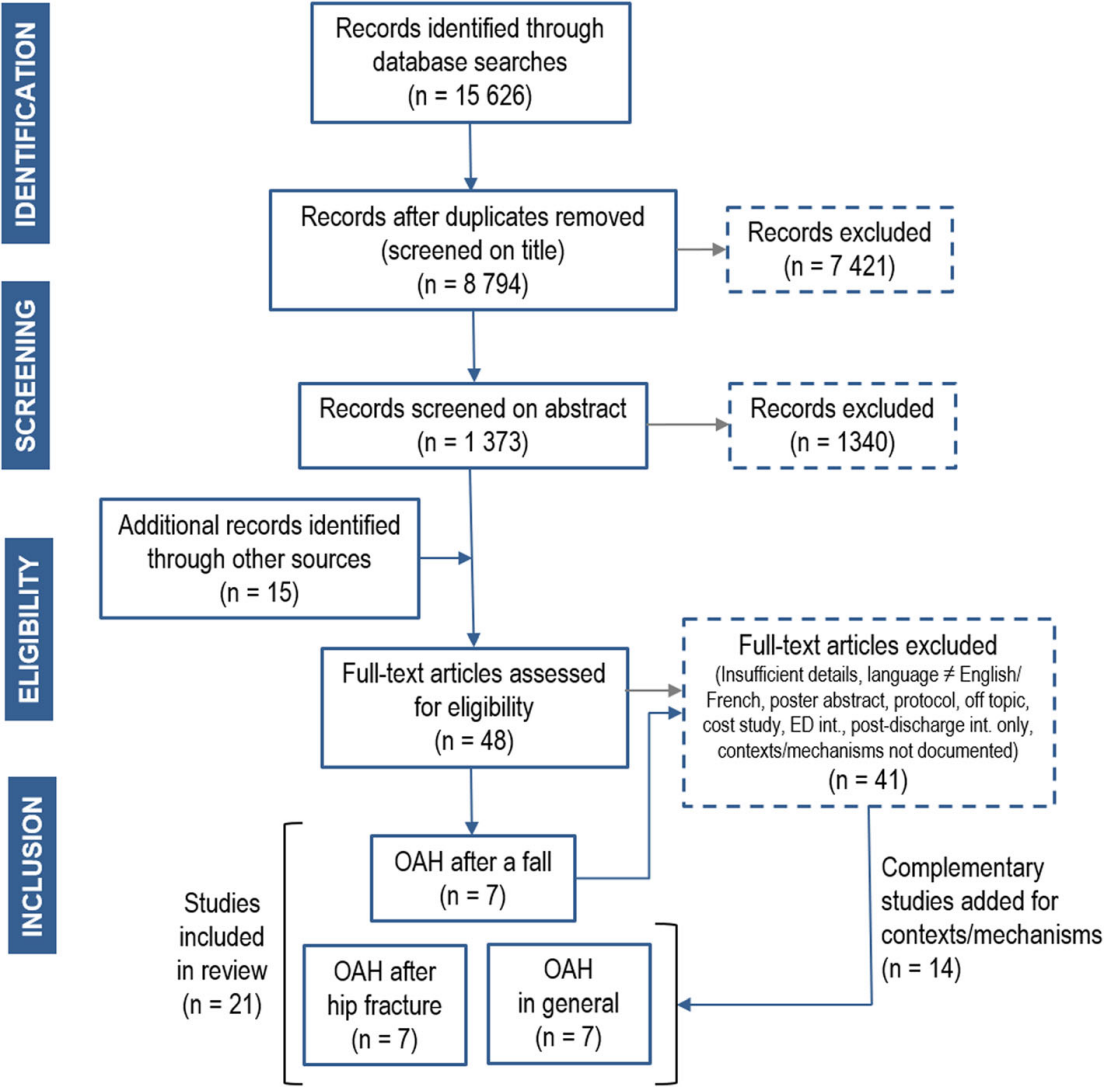
Document selection flowchart, OAH: older adults hospitalized, ED: emergency department, int: intervention

Figure 2 shows the flowchart for the selection of documents. Out of 8809 records identified (8794 through database searches after duplicates were removed and 15 through manual searches), the full text of 48 was assessed for final eligibility. Only seven documented contexts and mechanisms of interventions aimed at optimizing discharge planning for older adults hospitalized after a fall. Fourteen other documents related to older adults hospitalized for a hip fracture (*n* = 7) or hospitalized in general (n = 7) were added to enrich our understanding of contexts and mechanisms. Thus, a total of 21 documents were included in the synthesis.

Additional file 1 presents the descriptive of the selected documents. Comprising 20 scientific papers and one research report, the studies used different research strategies to investigate discharge planning. There were two Randomized Control Trials (RCT), four other quantitative studies, two mixed-methods processes, seven qualitative studies, one using both qualitative methods and a literature review, and five literature reviews. The latter were international (*n* = 5) while the others were conducted in different countries from almost every continent (North America 7, Europe 4, Oceania 3, South America 1, Asia 1). More than half the papers (57%; *n* = 12) were published between 2010 and 2014, nearly one third (29%; *n* = 6) since 2015, and 14% (*n* = 3) before 2010.

This realist synthesis led us to develop four ICMOs to better understand how and why the intervention components designed to improve discharge planning for older adults hospitalized following a fall might generate positive outcomes: 1) two-way communication between healthcare providers and patients/caregivers; 2) interprofessional communication within and across healthcare settings; 3) patient/caregiver individually tailored education on fall prevention; and 4) discharge planning coordination. Table 2 synthesizes the content of the four ICMOs.

**Table 2:** Synthesis of each Intervention-Context-Mechanism-Outcome (ICMO) configuration

INTERVENTION COMPONENTS (I)	CONTEXTS (C)	MECHANISMS (M)	OUTCOMES (O)
**ICMO-1: Two-way communication between healthcare providers and patients/caregivers**			
**Provide patients and families with individually tailored, complete and repeated information** [34] Regarding: health conditions, symptoms, how they evolve and how to manage them [9, 34] healthcare planning [9, 35–38] information seeking [9] Using: verbal and written communication [34] (adapted to each patient) [39] simple language [40] **Involve patients and families in healthcare and discharge planning** [9, 35, 36, 38, 41] Improve knowledge and understanding of families’ concerns, barriers and expectations to recovery [34, 41, 42] Have a comprehensive picture of the situation [9, 39]	**When** Throughout the discharge planning process - from admission [37, 43] to post-discharge (home) [37] - frequently [35] Communication on recovery time and risk management - before discharge [38, 41] **For whom** Some intrinsic characteristics may compromise communication [38, 44] - advanced age [38, 44] - high degree of frailty [38, 44] - living alone [38]	**For patients** ↑ understanding of how to balance risks safely [38] **For patients and families** ↑ knowledge of the illness/ injury [36] and how to manage it [9] ↑ adjustment of expectations regarding recovery [41] ↑ self-confidence, sense of control [38] and feeling of being prepared [37] ↓ stress and frustration [9, 34–36] ↓ confusion and tension between family members [37] **For healthcare providers** ↑ ability to address questions that patients do not know how to ask [41, 45]	↑ patients’ and families’ satisfaction [37, 41] ↑ patients’ recovery [38, 40] ↑ patients’ functional status [38, 40]
**ICMO-2: Interprofessional communication within and across healthcare settings**			
**Interprofessional communication and information sharing** accurate and effective [9, 36, 38–40, 45, 46] By using/doing: standardized routine for information exchange [9, 47] verbal and timely non formal communication [9] regular multidisciplinary meetings [9, 43, 47] complete handovers documenting fall risk [36, 46, 47] Web-based information system [9, 35, 39] (interoperable across care settings and available to all healthcare providers throughout the continuum of care) [39] clear boundaries for care (tasks and responsibilities) between all healthcare providers [35, 40]	**When** Throughout the healthcare continuum [9, 39] Between different healthcare settings [39] **Where** In a supportive organizational and management context (local and national levels) [44, 47]	↑ knowledge and understanding of healthcare providers regarding patients’ situations, and their own respective roles, tasks and responsibilities [9, 34, 46] ↓ redundancies, overlap, delays, inaccuracies, incompleteness, uncertainties regarding what has been done [34] ↓ losses of information across care settings [39] ↓ anxiety and frustration experienced by healthcare providers [39] ↓ time spent gathering information on patients [9]	↑ quality of care [9, 34, 39] ↓ hospital readmissions [9, 35] ↑ identification of patients at risk of falls [47]
**ICMO-3: Patient/caregiver individually tailored education on fall prevention**			
**Patients’ and caregivers’ education and training should:** Target real needs of patients at home [37, 48, 49] Teach possible prevention strategies and exercises to foster recovery [37, 38, 48, 49] Encourage and motivate patients to use these strategies and do the exercises [42, 48] Provide families with written educational material [48] **Caregivers’ education and training should also:** Cover patients’ medical condition, signs of complications, physical care requirements, medication, etc. (be prepared for “afterwards”) [37, 38]	**When** Before discharge [36, 49, 50] Reinforced education by follow up phone call post discharge [49] **For whom** Optimal in cognitively healthy patients [42, 48, 49] If patients cognitively impaired: caregivers’ education is essential [48]	**For patients and caregivers** ↑ awareness of fall prevention [37] ↑ recognition of near-falls [49] **For patients** ↑ knowledge of prevention strategies [42, 48] ↑ confidence and motivation to use them [42, 48] **For caregivers** ↑ knowledge of the illness/ injury makes them more resilient when providing care (↑ flexibility and abilities) [37, 38]	↓ fall risk [49, 50] ↓ negative psychological impacts on caregivers (burden) [37] ↑ safety in the care provided by caregivers at home [36] ↑ continuity for patients in transition from hospital to home [37]
**ICMO-4: Discharge planning coordination**			
**Designation of 1 pivotal healthcare provider (coordinator) to manage discharge planning** [35, 38, 39, 50]**:** Acting as the single regular contact point for patients [35] Coordinating a comprehensive intervention adapted to patients [50] **Tools to facilitate coordination:** Interdisciplinary worksheet to record all the barriers to a safe return home identified by all the healthcare providers [51] Web-based information system to integrate information from different providers and care settings [35, 50, 52]	**When** Throughout the discharge planning process - from admission to discharge [35, 36, 40, 51] - post-discharge (home) [36] **For whom** Older patients’ multiple comorbidities and medical complexities require extensive coordination as many healthcare providers are involved [35, 36, 41, 50, 53]	↑ stability and consistency through coordinator’s regular contacts with patients, families and professionals [34, 39] ↑ trust [39] ↑ identification, anticipation and alleviation of barriers experienced by patients [34, 35] ↑ communication and information sharing among healthcare providers and settings [53] ↑ identification and prioritization of patients’ needs [48] ↑ personal engagement from each healthcare provider and families over care [34]	↑ quality (continuity) of care [34, 39, 48, 53] ↑ patients’ quality of life (physical, psychological and social needs met) [48] ↑ patients’ satisfaction [52]

### ICMO-1: two-way communication between healthcare providers and patients/caregivers

#### Key findings

**Fig. 3:**
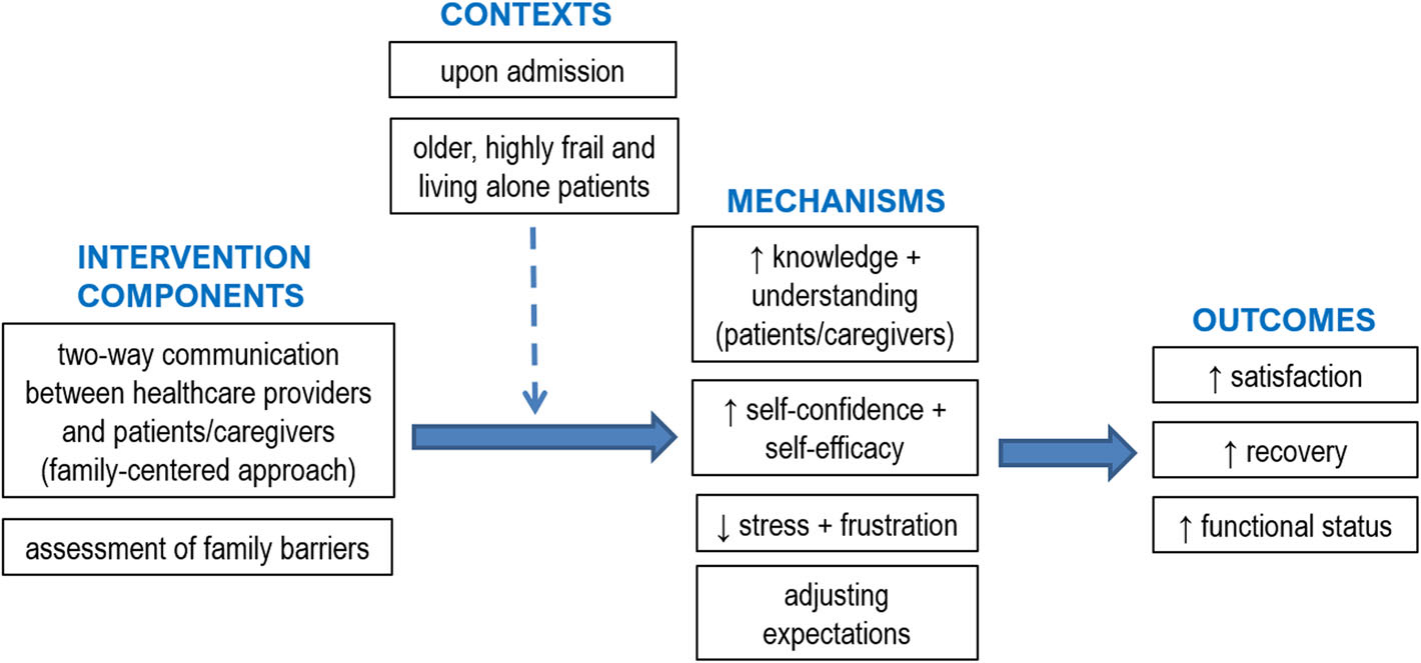
ICMO-1: Two-way communication between healthcare providers and patients/caregivers

The first ICMO (Figure 3) can be synthesized as follows: increase two-way communication between healthcare providers and families (patients and caregivers) regarding the patient’s health status, care provided and care planning, and address barriers experienced by families (I), when occurring early in the process (upon admission) (C), trigger a better patients’ understanding of how to manage risks safely, caregivers’ knowledge of the illness/injury and how to handle it, and families’ realistic expectations regarding recovery, self-confidence and self-efficacy (M), which produce an improvement of patient satisfaction, recovery and functional status (O).

#### Intervention components

Communication between healthcare providers and families (patients and caregivers) regarding the patient’s health status, care provided and care planning is one of the core components of these interventions (or suggested interventions to address gaps) to improve discharge planning for hospitalized older adults. Several studies report patients’ and caregivers’ frustration with communication during healthcare delivery [35, 36] as they often feel they have not received enough or appropriate information from healthcare providers [9, 35, 37, 38]. In their literature review of best practices for hospital discharge planning for frail older people, Bauer et al. [38] emphasized that the lack of communication between healthcare providers and patients/families was one of the main barriers to an effective discharge process.

Healthcare providers should give patients individually tailored, comprehensive, adequate and repeated information [34]. They should advise patients and caregivers how to manage the formers’ health conditions and symptoms and how to seek help [9], and they should provide families with written information [9, 34]. The way information is shared should be adapted to each patient [39]. Being informed should mean receiving not only information but also feedback, advice or reassurance from healthcare providers about the patient’s progress (after hip fracture, etc.) [34].

Families should also be included in planning care (current and follow-up) and discharge [9, 34–36, 38, 39, 41]. By engaging patients and their caregivers in discussions that recognize their perceptions of future risk, their concerns and the barriers to recovery they encounter or worry about, healthcare providers will know the right information to share with them [34, 41]. For example, many patients may experience a fear of falling when discharged home after a fall or hip fracture [34, 37, 41, 42]. In these cases, some advice from healthcare providers could be perceived as threatening their safety [34] and patients may avoid participating in activities that they could in fact do [41]. To ensure responsive interactions, it is crucial to have a better understanding of families’ concerns [34].

For fall-related hip fractures, some authors emphasize the importance of addressing patients’ expectations regarding recovery [42] and conducting a comprehensive assessment to develop a customized discharge plan [45]. Healthcare providers may use a preoperative classification system to assess patients and produce a more exhaustive, personalized recovery timeline for their patients [42]. Consulting relatives may also give them a more comprehensive view of the situation as family members may provide valuable information about the patient’s health [9, 39].

When talking with families, healthcare providers should use lay (non-technical) language and take the families’ views into consideration; otherwise, the latter may feel powerless and vulnerable [40].

#### Context

Many studies on discharge planning, including literature reviews, maintain that communication between healthcare providers and families is important throughout the discharge planning process, upon admission of patients to hospital [40] or within the next 24 to 48 h at most [38], and should be frequent [36]. A discussion on expected versus realistic recovery time and how to manage risk should take place before the discharge home [34, 42]. The most effective communication interventions started at an early stage and continued during the hospital stay and post-discharge phase [38]. According to some studies, the biggest communication gap occurs during the transition from hospital to home [34, 40].

Some intrinsic characteristics may also have an impact on communication. For example, with advanced age (85+) and a high degree of frailty, communication efficacy may be compromised [34, 43]. As the oldest old, frailest patients and those who live alone are more likely not to be informed, not remember being informed or be unable to understand the information provided and then take undue risks [34], adequate communication is particularly important in this context.

#### Mechanisms

Being well informed (i.e. provided with suitable information) improves patient understanding regarding how to balance risk safely [34] and increases caregiver knowledge of the illness/injury [37] and how to manage it [9]. This greater knowledge will reduce families’ stress and frustration [9, 35–37] and increase patients’ and caregivers’ self-confidence and self-efficacy [34], sense of control, self-care and symptom management after hospitalization [9]. To enhance their self-efficacy, patients need to acknowledge their progress and achievements [34]. When provided with adequate information, family members will be able to achieve a balance between making progress and potential dangers versus the use of protective strategies and following professionals’ instructions [34]. Conversely, a lack of information may cause anxiety and frustration, and make caregivers feel unprepared for discharge, which worsens their relationship with the patient [38]. Written information minimizes confusion and tension between family members [34].

Giving patients incomplete information leads to unrealistic expectations about recovery that are at odds with their lived experiences and makes them less engaged in their own recovery [42]. An information gap may also leave people with emotional struggles and misunderstandings, which induce them to take unnecessary risks [34]. Patients will adjust their expectations for their recovery based on the information received [42].

A better understanding by healthcare providers of patients’ experiences may further improve their capacity to address questions that patients do not know enough to ask, which increases the likelihood that the information patients receive is accurate and applicable to their specific condition [42, 44], and ensures appropriate and complete discharge instructions [41] and transitions [36].

#### Outcomes

All the mechanisms discussed above, triggered by better communication between healthcare providers and families, ultimately improve patient satisfaction [42], recovery, functional status and independence [34, 41]. A negative gap between expectations and reality results in patient dissatisfaction and disengagement [42]. In hip fracture studies, most patients were not satisfied with the information from healthcare providers regarding their surgery, the recovery process or their own progress [42]. Patients were dissatisfied because they took longer to recover than expected, they were not informed about which activities could help their recovery or the occurrence of unexpected post-operative complications, and they did not recover as well as expected [42]. In a review of discharge planning, Bauer et al. [38] noted that caregivers’ greater involvement in care planning (fostered by good communication) contributed to greater satisfaction with the process.

### ICMO-2: Interprofessional communication within and across healthcare settings

#### Key findings

**Fig. 4:**
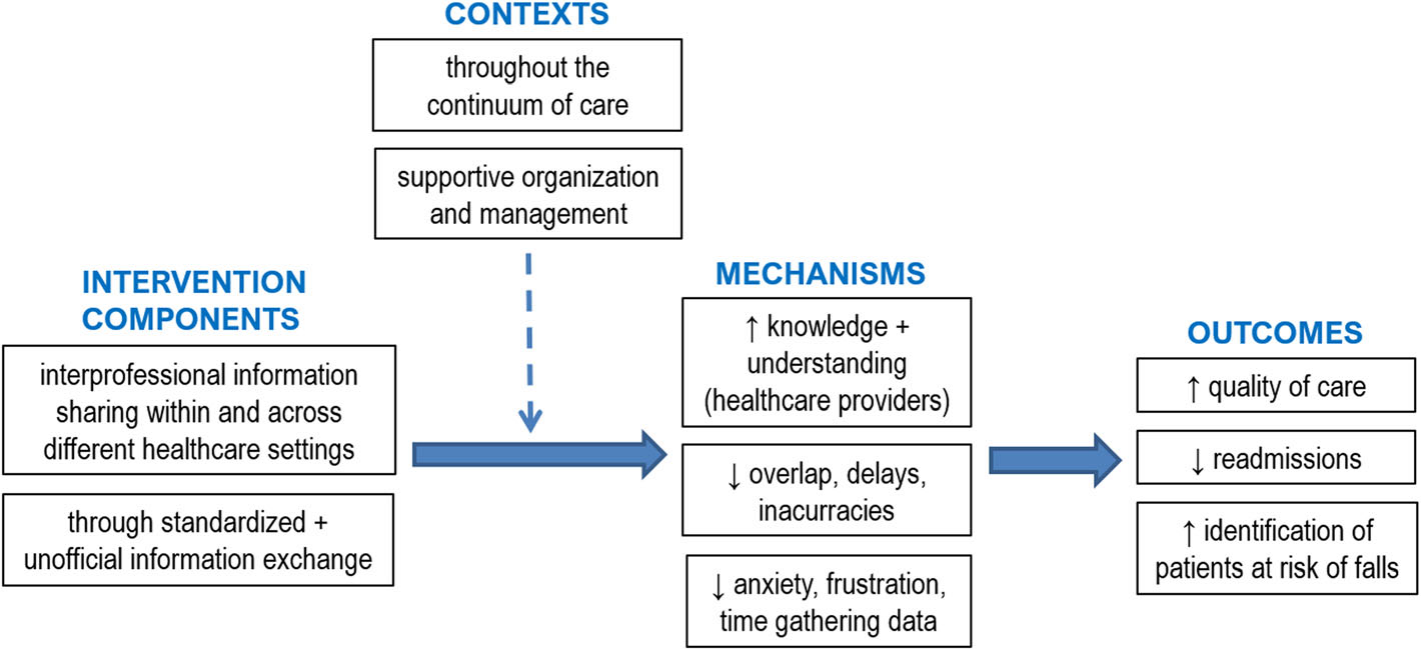
ICMO-2: Interprofessional communication within and across healthcare settings

ICMO-2 (Fig. 4) can be summarized as follows: interprofessional communication and information sharing within and across different healthcare settings through both standardized and unofficial information exchange (I), if supported by a favorable organizational and management context (C), trigger an improvement in healthcare providers’ knowledge and understanding of the patient’s situation and their respective roles and responsibilities, and less redundancies, delays and loss of information in patient handovers (M), which enhance the quality of care and identification of patients at risk of falls, and decreases the risk of hospital readmission (O).

#### Intervention components

In addition to communication with families, it is crucial to promote interprofessional communication and information sharing regarding the patient’s health status, care provided and care planning throughout the healthcare continuum and between different healthcare settings. As most hospitalized older patients have comorbidities, multiple concurrent diseases are addressed by numerous healthcare specialties and complex recoveries require various care settings; this means that the accuracy and effectiveness of information sharing between different healthcare providers are of the utmost importance [9, 36, 38–40, 45, 46]. In practice, however, this interprofessional communication is often lacking or interrupted [40, 46], especially between hospital and community healthcare providers [38, 40].

A standardized routine for exchanging information is advocated [9, 47], and a variety of communication channels are required (structured and unstructured, formal and informal). Multidisciplinary meetings should be held on a regular basis (which varies between different settings and according to foreseeable length of stay) to discuss treatment goals, the patient’s progress and discharge plan, and to standardize interprofessional communication [9, 43, 47]. Accurate and complete standardized handovers documenting fall risk would improve the quality of the transfer of information [36, 46, 47], which could be measured by the ISBAR (Identify, Situation, Background, Assessment and Recommendation) quality score (/5) [47]. However, patient handover documents or discharge summaries are often absent, incomplete or inaccurate, which leads to a communication gap between different healthcare settings or between acute and community healthcare providers [38, 40, 46].

It is practical to use a Web-based information system (or electronic health record) to facilitate exchanges regarding key patient information during handovers [9, 35, 39], as long as it is interoperable across care settings and available to all healthcare providers throughout the continuum of care [39]. Clear boundaries pertaining to the roles and responsibilities of each provider of patient care must be established among all healthcare providers, as they are often an area of misunderstanding [35, 40]. For example, in their review, Carroll and Dowling [40] reported that in two hospitals, the majority of nurses did not complete the discharge plan as they thought it was the case manager’s responsibility to do so. When interviewing healthcare providers involved in care transitions of older patients hospitalized for hip fracture, Toscan et al. [35] found that these professionals could not clearly describe the limits of their own responsibilities in the patient discharge care plan, nor those of other professionals within and across different care settings.

Verbal and timely informal communication between healthcare providers is also important throughout the process [9].

#### Context

Interprofessional communication should take place in a favorable organizational and management context. It is difficult for healthcare providers to change their fall prevention practices if the organization and the healthcare system (government and policymakers) do not support their efforts to communicate better [43, 47]. Optimized communication between different healthcare providers can only be achieved when there is strong, early engagement at the local and national organizational levels [47]. However, healthcare providers have reported that they often feel they are the only ones making an effort to change practices [47].

#### Mechanisms

Optimized interprofessional communication and information exchange increase healthcare team members’ knowledge and understanding of the patient’s situation and their respective tasks, roles and responsibilities (“who does what”) [9, 35, 46]. It is widely recognized that poor interprofessional communication generates redundancies, overlaps, delays, inaccuracies, incompleteness, uncertainties regarding what has been done versus what has to be done [35], and losses of information during transitions across care settings [39], which leaves healthcare providers feeling anxious and frustrated. Using an appropriate information system reduces the time spent gathering information about patients’ health condition, medical history and medication [9].

#### Outcomes

Healthcare providers’ greater knowledge and understanding of the patient’s situation and of what has been done by their counterparts in other fields, resulting from appropriate interprofessional communication, will improve the quality of transitional care received by older adults hospitalized following a fall [9, 35, 39] and decrease their risk of further hospitalization [9, 36]. Conversely, unnecessary hospitalizations and increased mortality and dependency are outcomes that result from a paucity of communication between healthcare providers [53]. It has been found that standardized communication between healthcare providers and improved quality of discharge information led to better identification of older patients at risk of falls [47].

### ICMO-3: patient/caregiver individually tailored education on fall prevention

#### Key findings

**Fig. 5:**
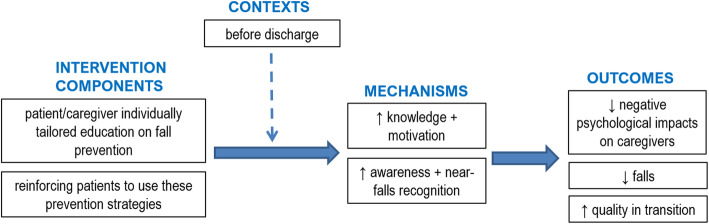
ICMO-3: Patient/caregiver individually tailored education on fall prevention

The third ICMO (Fig. 5) can be outlined as follows: providing older adults and their families with targeted fall prevention education and teaching, reinforcing and motivating patients to use these prevention strategies (I), when done before discharge (C), triggers an improved awareness regarding fall prevention, recognition of near-falls more easily and knowledge of and motivation to use prevention strategies (M), which will reduce the risk of falls post-discharge and negative psychological impacts on caregivers, and ensure a better transition from hospital to home for patients (O).

#### Intervention components

Next to communication, education encompasses another important group of intervention components used to optimize discharge planning for hospitalized older adults. These intervention strategies mainly involve fall prevention education tailored to the older adult [37, 48, 49]. For example, advocating a behavior change model of education, Hill et al. [48] reported positive outcomes with an intervention comprising an initial training session (during which a video was viewed and written material was given to the patient) followed by individual tailored in-person discussion sessions. In these individual sessions, the patient could discuss issues with the educator, and a phone call 2 weeks post-discharge reinforced what was learned. This intervention educated patients on fall prevention strategies as well as barriers and facilitators to using these strategies, fostered patients’ belief that they could use these strategies successfully, and provided cues for action. In their study, Sims-Gould et al. [42] noted that it was important for healthcare providers to not only teach patients how to do the exercises but also motivate them not to abandon their exercise program [42].

Different tools are used to assess and raise hospitalized patients’ awareness of the risk of falling. One study used home floor plans drawn by patients, combined with individual interviews with them, to identify potential fall hazards at home and develop a tailored education program targeting home and behavior modifications (e.g. reducing clutter, wearing appropriate footwear, adequate lighting) [49].

Some authors agree that healthcare providers should also provide caregivers with fall prevention [37, 38, 49] and health education [37]. As this is not always done, caregivers stress their need for more education concerning the patient’s medical condition and prognosis, signs of complications, physical care requirements, medications, and other specific care demands [38].

#### Context

Several studies have been conducted with cognitively intact patients [42, 48, 49]. However, with cognitively impaired patients, educational material and strategies should be adapted and caregivers’ input be included in the education process so that they can both acquire skills regarding how to handle daily impacts of major neurocognitive disorders [38]. Most authors agree that education interventions for patients and caregivers should take place at the hospital, prior to discharge [37, 48, 49]. One such intervention includes a follow-up call two weeks post-discharge to reinforce the education previously provided at the hospital [48].

#### Mechanisms

Education on fall prevention raises awareness in older people and their families [37] and contributes to a better recognition of near-falls, which is well known to be critical for preventing falls [49]. For patients, education gives them greater knowledge, confidence and motivation to engage in fall prevention strategies [42, 48]. For caregivers, greater knowledge of the illness/injury helps them take care of patients with more resilience, i.e. perform their caregiving role with increased flexibility rather than a rigid mindset [37] and with more proficiency [38]. Caregivers, on the other hand, often mentioned that they were not prepared for “post-hospitalization” [37] and this feeling of being unprepared made them anxious and frustrated [38].

#### Outcomes

By raising awareness and better recognition of near-falls, targeted fall prevention education for older adults and their families reduces the risk of falls [48, 49]. For example, participants in an intervention group (education on fall prevention strategies including a training video, written material and individual in-person discussions before discharge and follow-up phone call post-discharge) lowered their rate of falls to 5.4/1000 patients during the month post-discharge compared to 18.7 for the control group [48]. These participants were also more likely to plan how to resume to functional activities safely and to complete other targeted behaviors such as their home exercise program [48].

Educating caregivers reportedly leads to greater safety in the care they provide to their family members at home [37], less negative psychological impact on themselves and more continuity in patients’ transition from hospital to home [38].

### ICMO-4: discharge planning coordination

#### Key findings

**Fig. 6:**
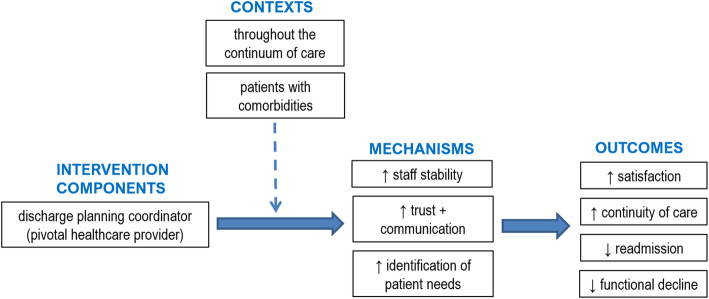
ICMO-4: Discharge planning coordination

The last ICMO (Fig. 6) is related to coordination and integration of healthcare services and can be synthesized as follows: the designation of a coordinator (pivotal healthcare provider) to manage patient care and act as the single contact point for the patient (I), throughout the continuum of care and, more importantly, for patients with comorbidities who have a large number of health professionals working with them (C), triggers enhanced staff stability and consistency of the information provided, trusting relationship between patients, families and professionals, and communication, information sharing and identification of patient needs (M), which improves patient satisfaction and the continuity of care and reduces the risk of hospital readmission and functional decline of the patient (O).

#### Intervention components

Communication and education strategies cannot achieve their goal if they are not well integrated and coordinated [40, 50, 53]; in fact, they are crucial components of integrated and coordinated healthcare services [35, 50].

One key component of a coordinated system is the designation of a pivotal healthcare provider to manage the care to be provided to older adults hospitalized following a fall [35, 38, 39]. This designated healthcare provider can act as the single contact point for patients [35], coordinate comprehensive and customized interventions for this frail population [50], and address the needs of families with input from a multidisciplinary team [38]. A systematic review of the literature on osteoporosis care in orthopedic environments revealed that 65% of the healthcare systems analyzed included a dedicated coordinator who acted as the link between the orthopedic team, osteoporosis and fall services, the patient and the primary care physician [50]. The assigned coordinator may be called a system coordinator [35], discharge coordinator [38] or case manager [39, 50].

Different tools are used to optimize the coordination of transitional care. For example, Dedhia et al. [51] used an interdisciplinary worksheet to record all the barriers to the patient’s safe return home identified by each of the different professionals part of the Safe STEPS (Safe and Successful Transition of Elderly Patients Study) Intervention Program. In addition to facilitating communication between healthcare providers, an information system or electronic medical records would also help to achieve integration of patient information from different healthcare providers and settings [35, 50, 52] but this remains a challenge for many healthcare systems [52].

#### Context

Many studies agree that integration and coordination of healthcare should take place throughout the process, from patient admission to discharge home [35, 36, 40, 51], and during the follow-up process [36]. However, in many healthcare systems, providers still lack the time and resources to coordinate the care and discharge of older patients effectively [35, 53].

Because of multiple comorbidities and medical complexities, older patients require more extensive healthcare coordination because of the larger number of professionals working with them [35, 36, 41, 50, 53]. For example, patients with a hip fracture need surgical, geriatric, rehabilitation and psychosocial services to be integrated [36]. When a patient transitions from one healthcare setting to another, the healthcare teams responsible for discharge are often not the same [35], which poses a challenge when trying to coordinate services.

#### Mechanisms

Staff stability and consistency of the information provided in the healthcare delivered are reinforced when the coordinator provides a single regular contact point for patients, families and professionals [35, 39]. Relationships of trust are established between team members, external providers, patients and families [39]. Coordination and integration help healthcare providers to identify, anticipate and alleviate barriers experienced by patients [35, 36] and help the multidisciplinary team to make a more appropriate use of resources [53]. Integrated information systems would improve communication and information sharing between healthcare providers and healthcare settings [52]. Without integration and coordination, the size of the healthcare team may grow with the complexity of older patients’ medical needs and lessen the personal engagement of each healthcare provider and the family in providing care [35]. A lack of integration and coordination of healthcare may also lead to poor communication between healthcare providers, and less awareness and inappropriate prioritization of patients’ needs [53].

#### Outcomes

By reinforcing the stability of staff and consistency of the information provided in the delivery of care, the coordination and integration of healthcare services for older adults hospitalized after a fall enhance the quality and continuity of care provided to patients [35, 39, 52, 53]. According to Khatib et al. [53], if patient needs are not properly identified and prioritized, the healthcare provided will be fragmented and of lower quality. Quality healthcare contributes to a better quality of life for patients as their physical, psychological and social needs will be met [53].

Using an interdisciplinary team worksheet coordinated by a case manager skilled in discharge planning, Dedhia et al. [51] found an increase in the proportion of patients with high-quality transitions home (patient satisfaction measured by Coleman’s Care Transition Measures) from 68% before the intervention to 89% after, and a lower rate of readmission (22 to 14%).

## Discussion

This realist synthesis sheds light on contexts and underlying mechanisms of the outcomes of intervention components aimed at optimizing discharge planning for older adults (65+) hospitalized following a fall. Four ICMOs were developed and grouped into three highly interrelated domains of discharge planning: communication, education and coordination.

### ICMO-1: two-way communication between healthcare providers and patients/caregivers

The first ICMO emphasizes the importance of not only informing families but also involving them in care and discharge planning. In this patient-centered approach, making joint decisions with families is often suggested. Healthcare providers should offer possible options, not impose a decision. A balance should be achieved between informing and deciding with families without disempowering them. Offering patient-adapted options to guide informed decision-making is relevant but may be ethically difficult to do. Communication should be a two-way street as healthcare providers should give enough information to foster dialogue with patients and caregivers. Because it would be relevant to have families generate answers by themselves and target interventions acceptable to them, it is important for healthcare providers to offer options. However, the literature does not shed any light on exactly how to operationalize families’ integration in making decisions about discharge planning. Authors agree that communication should begin upon patient admission but more research is needed to determine specifically what should be said (information to provide and questions to ask to elicit informative answers from families) and when, as patients’ perceptions and needs can change depending on when the information is shared [54, 55].

Family members play a central role in communication as they provide information about the patient’s health, habits and values, ask for appropriate health services and support the patient with self-care [9, 39]. However, some may feel burdened and pressured to take on extensive responsibility for the patient [9]. As argued by Funk [56], to prevent family members from feeling that the whole burden lies on their shoulders, they should be supported during the transition and provided with tools to navigate in the system. Despite relatives’ essential role, their presence may not always be helpful: 13% of older adults who received assistance with one or more activity of daily living reported that this aid was only somewhat or not at all reliable [57]. Patients and caregivers may not share the same opinion regarding which treatment option is best and thus be inclined to make different decisions [58]. Family involvement in planning care is often valued but healthcare providers must be vigilant regarding potentially problematic situations [58].

### ICMO-2: Interprofessional communication within and across healthcare settings

Since discharge information from one care setting becomes admission information for another [39], a key element of interprofessional communication is to make sure that the shared information is understood correctly by the recipient. Mansah [59] highlights the importance of communication in care transitions for the older adults and discusses the theory of “planned communication”, which takes into consideration the receiver of the message during the transition.

Defining clear boundaries for the roles and responsibilities of the different healthcare providers involved in discharge planning for older adults has been targeted as an important step in optimizing interprofessional communication [9, 35, 40, 46]. Clarifying roles so that they are complementary is important but challenging. Duplication of some interventions at different times and in various settings can be beneficial if done at the right time and in the right context to prevent service gaps. However, a flexible approach is needed to keep in mind what is best for the patient.

### ICMO-3: patient/caregiver individually tailored education on fall prevention

While some hospitalized patients fear falling when thinking about their discharge home [34, 36, 37, 47, 60], many older adults do not worry about it (believing that fall prevention is for others), even if they were hospitalized following a fall [61]. According to Meyer et al. [61], fall prevention is not a priority for older patients admitted to hospital after a fall if they have another acute medical condition, which becomes the priority for them. These situations pose a challenge as they increase the difficulty of making evidence-based fall prevention strategies relevant and a priority for older adults [61]. It is crucial to educate patients on the possible severity of the consequences (including death) of a possible fall, while highlighting what can be done to minimize the consequences, with few changes in their daily lives.

The theory of planned communication [59] can also be applied to education since the way information is taught and the content of the message should be adapted to the recipients’ characteristics or specific needs. An example of adaptation is using pictograms and images showing good practices after a hip replacement to educate patients with a low level of literacy [62].

### ICMO-4: discharge planning coordination

Most of the time, having a coordinator induces greater trust, more familiarity and less anxiety for patients, caregivers and healthcare providers, but a major challenge is keeping the same coordinator throughout the process to maintain stability. Sustaining these bonds of trust in healthcare contexts where staff turnover [63] and a lack of resources [35] are common can be even more challenging. Could the continuity of transmitted information resulting from coordination minimize the impacts of personnel changes? In difficult healthcare contexts, it is essential to optimize information transmission from hospital to home to ensure that patients do not have to repeat their story and to avoid redundancies or duplication.

Another coordination challenge is formalizing two-way communication channels with other healthcare providers, i.e. anchoring agendas to ensure verbal information-sharing. Regular phone calls between hospital and community-based clinicians may create a win-win relationship where each professional feels that it is possible to provide and receive information. As mentioned by Zurlo and Zuliani [17], the ability to communicate among healthcare providers involved in discharge planning for older patients inside and outside hospital settings is essential to ensure an effective process. Creating a feedback loop concerning patient outcomes after discharge may also enhance interprofessional communication [64] and improve clinicians’ practices with future patients.

Currently, many healthcare systems cannot afford a dedicated coordinator for every patient. This may raise the ethical issue of who should be prioritized to improve the safety and quality of life of a more vulnerable subgroup without limiting access to services for less vulnerable patients. This distributive justice value should not be overlooked in aging populations where needs are increasingly complex and resources are limited [65, 66].

### Limitations

Because of the subjective and interpretative nature of this approach, the results of this realist synthesis reflect the analysis and inferences made by those who examined the information in the literature reviewed [67, 68]. Other reviewers might have generated different ICMOs. It is clear from the scientific literature that realist syntheses are not easy to reproduce [28]. However, the grid developed and used by the research team to analyze the documents ensured consistency in data extraction. Also, the ICMOs were developed based on an iterative process involving three team members and regular discussions between them. Input from knowledge users was also considered to optimize their clinical relevance.

The small number of documents included in this realist synthesis (*n*=21) may limit the scope of the results. However, the decision to retain only studies that enriched our understanding of mechanisms and contexts (and reject those that did not provide information on these aspects) meant that we worked with a relevant corpus of data. When conducting a realist synthesis, the relevance of documents takes priority over the number [28].

Unfortunately, contextual factors are often not sufficiently detailed in the existing literature, which limits reviewers’ ability to clearly and exhaustively identify these factors and how they influence the relationships between intervention mechanisms and outcomes [69–71]. However, our approach enabled us to develop preliminary theories which will have to be empirically tested to validate and strengthen the ICMO configurations.

## Conclusion

This realist synthesis is a first step in developing preliminary theories with a view to making recommendations for the implementation of best practices related to discharge planning for older adults hospitalized following a fall. As transitions are critical points with potential communication gaps, coordinated interventions are vital to support the transition from hospital to home. Changes in behaviors must be “endorsed” by both clinicians and managers. Clinicians may not want to make these changes alone (for accountability reasons), which means that more resources are required in the short term. Also, these changes may not be initiated by managers alone as clinicians need to ensure their sustainability. Considering the organizational challenges, clinicians’ use of simple tools (such as pictograms and drawings), combined with computer-based communication channels, may foster the return home safer for frail patients by providing interventions tailored to their needs, habits and values.

The next step is to refine and test these preliminary theories against empirical data. More specifically, further studies are needed to document exactly when in the continuum of transitional care (pre- or post-discharge) and where (hospital, home or both) interventions should take place. Implementation in acute or post-acute care contexts could have different outcomes, which warrants further investigation as longer hospital stays may leave more time for communication and education. More information is also needed on how communication and education should be organized, e.g. extent to which a follow-up phone call or mobile videoconference could replace a home visit when planning the discharge home of hospitalized older adults after a fall. To our knowledge, there is insufficient evidence regarding these aspects to support a transition of these patients that is not only effective but also efficient, i.e. has a positive impact but requires less time and resources. While fostering the safe return home of these patients, empirically testing our preliminary theories will help to develop effective interventions throughout the continuum of transitional care, enhance patients’ health and reduce the economic burden of avoidable care.

## Supplementary Information

**Additional file 1. **Descriptive of the selected documents (*n*=21).

## Data Availability

The datasets analysed during the current study are available from the corresponding author on reasonable request.

## Abbreviations

ICMO: Intervention-Context-Mechanism-Outcome
ISBAR: Identify, Situation, Background, Assessment and Recommendation
RAMESES: Realist And Metanarrative Evidence Syntheses: Evolving Standards
RCT: Randomized Control Trials
STEPS: Safe and Successful Transition of Elderly Patients Study
