# Supplementary material for: Understanding the positive outcomes of discharge planning interventions for older adults hospitalized following a fall: a realist synthesis
Source: BMC Geriatr. 2021 Jan 29;21:84. doi: 10.1186/s12877-020-01980-3 (PMC7844968; doi:10.1186/s12877-020-01980-3)
Supplement: Supplementary file 1 — Additional file 1. Descriptive of the selected documents (n=21). [file 12877_2020_1980_MOESM1_ESM.docx]

**Additional file 1: Descriptive of the selected documents (n=21)**

| **DESCRIPTIVE** | **N** | **REFERENCE NUMBERS** |
| --- | --- | --- |
| **Focus** |  |  |
| Older adults hospitalized after a fall | 7 | 35, 36, 38, 40, 41, 46, 50 |
| Older adults hospitalized for a hip fracture | 7 | 9, 34, 39, 42, 44, 47, 51 |
| Older adults hospitalized in general | 7 | 37, 43, 45, 48, 49, 52, 53 |
| **Manuscript type** |  |  |
| Scientific paper | 20 | 9, 34-44, 46-53 |
| Research report | 21 | 45 |
| **Methodology (research strategy)** |  |  |
| Randomized Control Trial | 2 | 49, 50 |
| Other quantitative study | 4 | 44, 46, 52, 53 |
| Mixed-methods | 2 | 45, 47 |
| Qualitative | 7 | 9, 34, 36, 38-41 |
| Literature review | 5 | 37, 42, 43, 48, 51 |
| Qualitative + literature review | 1 | 35 |
| **Territorial coverage** |  |  |
| North America | 7 | 34, 35, 39, 40, 41, 52, 53 |
| South America | 1 | 36 |
| Europe | 4 | 9, 38, 44, 45 |
| Oceania | 3 | 46, 47, 49 |
| Asia | 1 | 50 |
| International | 5 | 37, 42, 43, 48, 51 |
| **Publication year** |  |  |
| < 2010 | 3 | 37, 43, 52 |
| 2010-2014 | 12 | 9, 34, 35, 38-40, 42, 45, 47, 49, 51, 53 |
| ≥ 2015 | 6 | 36, 41, 44, 46, 48, 50 |
